# Immunomodulation during prolonged treatment with combined interleukin-2 and interferon-alpha in patients with advanced malignancy.

**DOI:** 10.1038/bjc.1993.29

**Published:** 1993-01

**Authors:** A. von Rohr, A. K. Ghosh, N. Thatcher, P. L. Stern

**Affiliations:** CRC Department of Medical Oncology, Paterson Institute for Cancer Research, Christie Hospital and Holt Radium Institute, Manchester, UK.

## Abstract

Treatment with combined IL-2 and alpha-IFN has resulted in synergistic antitumour efficacy in animal studies. The mechanisms responsible for this synergy remain unclear. In this study, several immune parameters which might be involved in mediating antitumour activity have been monitored serially in 15 patients with advanced malignant melanoma or renal cell cancer during treatment with concurrent IL-2 and alpha-IFN. Both drugs were given subcutaneously in low to moderate (outpatient) dosages but for a prolonged duration. This treatment resulted in remarkable immunomodulation. In vivo induction of cytotoxicity against K562 and Daudi target cells was consistently seen, and percentages of peripheral blood cells expressing CD 25 (IL-2 receptor) and CD 56 (Leu-19) increased. In vitro proliferation of lymphocytes in response to IL-2 was enhanced during the treatment periods, whereas spontaneous proliferation was inhibited. Moreover, correlations between immune parameters and subsequent clinical responses were present in the early phase of the study. Cytotoxicity levels generated in vivo as well as the percentage of CD 56+ lymphocytes were higher in patients who responded to treatment than in non-responders. In contrast, responders had lower levels of CD 25+ cells. These findings indicate that it might be possible to select patients who are likely to benefit from prolonged immunotherapy.


*Br. J. Cancer* (1993), **67**, 163–171 © Macmillan Press Ltd., 1993

### Immunomodulation during prolonged treatment with combined interleukin-2 and interferon-alpha in patients with advanced malignancy

A. von Rohr[1], A.K. Ghosh[2], N. Thatcher[1] & P.L. Stern[2]

[1]*CRC Department of Medical Oncology, and* [2]*CRC Department of Immunology, Paterson Institute for Cancer Research, Christie Hospital and Holt Radium Institute, Manchester, UK.*

**Summary** Treatment with combined IL-2 and α-IFN has resulted in synergistic antitumour efficacy in animal studies. The mechanisms responsible for this synergy remain unclear. In this study, several immune parameters which might be involved in mediating antitumour activity have been monitored serially in 15 patients with advanced malignant melanoma or renal cell cancer during treatment with concurrent IL-2 and α-IFN. Both drugs were given subcutaneously in low to moderate (outpatient) dosages but for a prolonged duration. This treatment resulted in remarkable immunomodulation. *In vivo* induction of cytotoxicity against K562 and Daudi target cells was consistently seen, and percentages of peripheral blood cells expressing CD 25 (IL-2 receptor) and CD 56 (Leu-19) increased. *In vitro* proliferation of lymphocytes in response to IL-2 was enhanced during the treatment periods, whereas spontaneous proliferation was inhibited. Moreover, correlations between immune parameters and subsequent clinical responses were present in the early phase of the study. Cytotoxicity levels generated *in vivo* as well as the percentage of CD $56^+$ lymphocytes were higher in patients who responded to treatment than in non-responders. In contrast, responders had lower levels of CD $25^+$ cells. These findings indicate that it might be possible to select patients who are likely to benefit from prolonged immunotherapy.

Interleukin-2 (IL-2) and interferon-alpha (α-IFN) are cytokines with independent antitumour activities in animal models as well as in some human malignancies including malignant melanoma (MM) and renal cell cancer (RCC). The exact mechanisms by which IL-2 and α-IFN cause tumour regression *in vivo* are as yet unknown; from experimental studies, however, both agents are known to have a number of functions which possibly contribute to their antitumour efficacy.

IL-2 has initially been described for its capability to support the growth of T lymphocytes. In addition, IL-2 plays a central role in the regulation of cell-mediated non-major histocompatibility complex (MHC)-restricted cytotoxicity. IL-2 augments the cytolytic activity of natural killer (NK) cells (Henney *et al.*, 1981) and induces the generation of lymphokine-activated killer (LAK) cells (Grimm *et al.*, 1982). NK cells are lymphocytes exhibiting a spontaneous cytotoxicity against some tumour cells and tumour cell lines (Robertson & Ritz, 1990). Incubation of peripheral blood lymphocytes (PBL) in IL-2 for at least 3 days results in the generation of LAK cells which are capable of lysing NK resistant tumour cell lines and fresh tumour cells (Grimm *et al.*, 1982) but show only minimal cytotoxicity against normal cells (Sondel *et al.*, 1986). It has been shown in human and animal studies that significant LAK activity can also be generated *in vivo* by treatment with appropriate IL-2 regimens.

The cell populations mediating NK and LAK cytotoxicity are functionally defined and composed of phenotypically heterogeneous lymphocyte subpopulations. The surface antigen Leu-19 (CD 56) is expressed by virtually all human NK effectors (Lanier *et al.*, 1986) although they are heterogeneous for the expression of additional markers; moreover, less than 5% of T lymphocytes, monocytes, and neutrophils are Leu-$19^+$ (Robertson & Ritz, 1990). Leu-19 is therefore used most extensively as NK cell marker. In addition, Leu-19 expression has been demonstrated in the majority of LAK effectors, and there is considerable evidence that LAK activity is largely mediated by IL-2 activated NK cells (Ortaldo *et al.*, 1986; Phillips & Lanier, 1986; Weil-Hillman *et al.*, 1989).

Correspondence: A. von Rohr, Central Hematology Laboratory, Inselspital, CH-3010 Berne, Switzerland.
Received 13 March 1992; and in revised form 3 August 1992.

IL-2 also stimulates proliferation of various lymphocyte subpopulations (Bich-Thuy *et al.*, 1986) including NK cells (Trinchieri *et al.*, 1984; Yamada *et al.*, 1987). After *in vivo* administration of IL-2, considerable changes in the phenotypic composition of circulating lymphocytes are usually observed, including increased percentages of cells expressing the IL-2 receptor (CD 25) and Leu-19 (Ellis *et al.*, 1988; Lotze *et al.*, 1987).

α-IFN is known to augment the cytotoxic activity of NK cells (Herberman *et al.*, 1982). Moreover, α-IFN has direct antiproliferative effects on normal and neoplastic cells (Czarniecki *et al.*, 1984; Fidler *et al.*, 1987). In tumour cells, α-IFN enhances the expression of cell surface molecules including tumour-associated antigens and class I MHC antigens (Giacomini *et al.*, 1984; Weber & Rosenberg, 1988) and is capable of promoting a partial reversal of the malignant phenotype (Hicks *et al.*, 1981).

In animal studies, the administration of combined IL-2 and α-IFN resulted in synergistic antitumour efficacy (Brunda *et al.*, 1987; Cameron *et al.*, 1988; Iigo *et al.*, 1989; Kim *et al.*, 1989; Rosenberg *et al.*, 1988). The mechanisms responsible for this *in vivo* synergy remain largely speculative, although the interactions between IL-2 and α-IFN have been investigated in a number of *in vitro* studies. These experimental data clearly show that combination of α-IFN and IL-2 results in a more than additive augmentation of NK activity (Brunda & Davatelis, 1985). In contrast, *in vitro* generation of LAK cells seems to be modulated by α-IFN in a complex manner. Culture of PBL in IL-2 and α-IFN may result in either enhanced or impaired LAK activity depending on experimental conditions such as timing of α-IFN addition, sequential or concurrent exposure of PBL to IL-2 and α-IFN, α-IFN concentration, and duration of the culture period (Brunda *et al.*, 1986; Chikkala *et al.*, 1990; Di Raimondo *et al.*, 1987; Sone *et al.*, 1988; Tokuda *et al.*, 1989). In addition, α-IFN may also affect tumour cells resulting in either increased or reduced susceptibility to NK and LAK cell lysis depending on the experimental conditions and the target cells used (Di Raimondo *et al.*, 1987; Greenberg *et al.*, 1984; Metha *et al.*, 1991; Trinchieri *et al.*, 1981).

In contrast to an abundance of *in vitro* studies, only limited data exist on immunological effects of combined IL-2 and α-IFN occurring *in vivo*. In the present study, we have serially monitored *in vivo* generated cytotoxicity against K562 and Daudi target cells, lymphocyte proliferation, and expression of selected lymphocyte surface markers in cancer patients undergoing treatment with combined IL-2 and α-IFN. Whilst detailed clinical data of this study will be presented elsewhere, we report here on the immunological aspects.

### Material and methods

### Patients and treatment

The immunological data analysed in this paper were generated by 15 patients entered into a phase II trial. All patients had advanced, progressing MM or RCC. Treatment consisted of concurrent interleukin-2 (IL-2, Proleukin®; kindly provided by EuroCetus Ltd.*), and interferon-alpha-2a (α-IFN, Roferon®-A; Hoffmann-LaRoche). Both drugs were administered subcutaneously on an outpatient basis. IL-2 was given five times a week (Monday to Friday) in fixed single doses of $18 \times 10^6$ IU, and α-IFN was given three times a week (Monday, Wednesday, Friday) in fixed single doses of $3 \times 10^6$ IU, for three successive weeks, followed by a rest period of 2 weeks (Figure 1). In case of tumour remission or stable disease, treatment was continued to a maximum of four cycles with unchanged drug dosages. All patients included in this analysis received at least one full treatment cycle and were evaluable for clinical response. Complete or partial remissions (CR/PR) were seen in three patients, no change (NC) of tumour parameters in four patients, and progressive disease (PD) in eight patients. All three responders had MM.

### Patient monitoring and sample preparation

Thirty to forty ml of heparinised blood were drawn from patients on days 1 (pretreatment), 5, 12 and 19 of each treatment cycle (sampling days; see Figure 1) for serial assessments of cytotoxicity, surface marker analysis, and proliferation assays. PBL were separated by Ficoll-Hypaque density gradient centrifugation and resuspended in RPMI 1640 tissue culture medium supplemented with L-glutamine (2.0 mM), ampicillin (200 mg $l^{-1}$), streptomycine (250 mg $l^{-1}$) and 10% (v/v) heat-inactivated foetal calf serum (subsequently referred to as 'medium'). Viable cells were then counted by the trypan blue exclusion method. Excess cells were cryopreserved by controlled rate freezing in 10% dimethylsulfoxide and stored in liquid nitrogen.

### Cytotoxicity assay

A 4 h $^{51}$Cr-release assay was applied to test cytotoxic activities, using fresh PBL as effector cells and the two tumour cell lines K562 and Daudi as target cells. Details of the technique have been described previously (Ghosh et al., 1989). Briefly, targets were labelled with 0.1 mCi $^{51}$Cr/$10^6$ cells for 1 h at 37°C, washed twice, and then incubated for 30 min at 37°C with medium to reduce spontaneous chromium release. After two further washings, the targets were resuspended in medium for a concentration of $5 \times 10^4$ cells $ml^{-1}$. One hundred μl of effector cells in appropriate dilutions and an equal volume of labelled targets were added to LP2 tubes resulting in effector:target (E:T) ratios of 100:1, 40:1, 20:1 and 10:1. Maximum chromium release was obtained by additing 100 μl of 1% Tween 20 detergent to the targets instead of effectors, and spontaneous release by adding 100 μl of medium. After short centrifugation (600 r.p.m., 5 min), the LP2 tubes were incubated for 4 h (37°C, 5% $CO_2$). All assays were done in triplicate, and no IL-2 was present during the incubation period.

Following incubation, 100 μl of supernatant were removed to LP3 tubes, and the radioactivity of supernatant and remaining pellet was counted in a gamma counter. Cytotoxicity results are expressed as percentage specific cytotoxicity, which was calculated according to the formula

$$\%\ \text{cytotoxicity} = 100 \times \frac{\text{experimental c.p.m.} - \text{spontaneous c.p.m.}}{\text{maximum c.p.m.} - \text{spontaneous c.p.m.}}$$

Statistical analysis was done for serial cytotoxicity results, as assessed on the four sampling days per treatment cycle, as well as for peak cytotoxic activity which was defined as maximum cytotoxicity reached at any occasion during one treatment cycle.

**Figure 1** Treatment plan and collection of blood samples.

*Specific activity: $18 \times 10^6$ international units (IU) per mg of protein. 1 Cetus unit = 6 IU (all IL-2 dosages are given in IU).

### Lymphocytes phenotype analysis

In nine patients (2 CR/PR, 1 NC, 6 PD) enough PBL were left for serial analysis of two surface markers during the first treatment cycle. This was performed by an indirect immunofluorescence method on cryopreserved and thawed cells. Monoclonal antibodies used were DAKO-IL2-R (Dakopatts, Denmark), directed against the 55 kDa polypeptide chain of the IL-2 receptor (CD 25, Tac antigen); and Leu-19 (Becton Dickinson), directed against the NK cell marker CD 56 (NKH1). A FITC-conjugated rabbit anti-mouse immunoglobulin antibody (Dakopatts, Denmark) was used as second step reagent, and the percentage of fluorescence positive cells was assessed by FACS analysis. Results obtained serially on the sampling days as well as peak percentages, which were defined as highest relative numbers of fluorescence positive cells at any occasion during one treatment cycle, were subject to statistical analysis.

### PBL proliferation assay

Spontaneous (unstimulated) and stimulated proliferation of PBL was serially tested, the former by culture of cells in medium alone, the latter by incubation in phytohemagglutinin (PHA; Wellcome) or IL-2. Fresh PBL were seeded in 96-well U-bottomed microtiter plates under sterile conditions. Each well contained $2 \times 10^5$ cells in 200 μl of medium with or without the stimulant. PHA was added for final concentrations of 1.0 μg $ml^{-1}$, 0.1 μg $ml^{-1}$, or 0.01 μg $ml^{-1}$, and IL-2 was added for final concentrations of 1,200 IU $ml^{-1}$, 240 IU $ml^{-1}$, or 120 IU $ml^{-1}$. All tests were done in triplicate. Cultures were incubated for 96 h at 37°C in a humidified atmosphere containing 5% $CO_2$. For the last 4 h, each well was pulsed with 1 μCi $^3$H-thymidine. Cultures were harvested semiautomatically onto fibreglass filters, and filters were dried overnight at room temperature. Radioactivity was counted by liquid scintillation. Results for spontaneous proliferation are given as counts per min (c.p.m.), and results for stimulated proliferation are expressed as a stimulation index (SI) in order to take account of fluctuations in spontaneous proliferation. The SI was computed as follows:

$$\text{SI} = \frac{\text{c.p.m. (stimulated)}}{\text{c.p.m. (spontaneous)}}$$

Peak proliferation was defined as maximum proliferation (c.p.m. or SI) reached at any occasion during one treatment cycle.

### Statistical analysis

Statistical analysis was done by non-parametric tests. The Wilcoxon matched-pairs signed-ranks test (hereafter referred to as Wilcoxon test) was used for paired samples. Data from patients in different response groups were compared by the Kruskal-Wallis test or the Mann-Whitney test as appropriate. All $P$-values reported are 2-tailed, and $P<0.05$ was considered to indicate statistical significance.

### Results

### In vivo *cytotoxicity*

*Pretreatment cytotoxicity* Median pretreatment cytotoxic activities against both K562 and Daudi targets are given in Tables I and II for each of the 4 E:T ratios tested. An activity of >10% against the K562 target was observed in 79% of patients before treatment. One patient (who subsequently responded to therapy) had an unusual high pretreatment anti-K562 activity of 74% for the E:T ratio of 100:1, and values up to 46% were found in the remaining patients. A maximum pretreatment cytotoxicity of 13% against the Daudi target was seen for the same E:T ratio.

*Cytotoxicity during the first treatment cycle* *In vivo* induction of cytotoxicity against both K562 and Daudi targets was consistently seen after initiation of IL-2/α-IFN therapy. At an E:T ratio of 100:1, peak anti-K562 activities obtained by individual patients ranged from 28% to 74% (median 50%). Individual peak activities against Daudi targets ranged from 7% to 58% (median 35%), with only one patient not exceeding the 20% limit. The differences between pretreatment and peak activity were statistically significant for both targets at each of the four E:T ratios tested (see Tables I and II).

Figures 2a and 2b show that cytotoxicity was markedly augmented by day 5, the increase since day 1 being statistically significant for both anti-K562 and anti-Daudi activities ($P\leqslant0.013$ for the K562 target, and $P\leqslant0.009$ for the Daudi target, at each of the four E:T ratios; Wilcoxon test). A continuing increase of cytotoxicity levels was seen during the second treatment week before a plateau phase was reached during the third week.

*Relationship between cytotoxicity and response* Cytotoxicity results were compared between patients grouped according to

**Table I** Pretreatment and peak cytotoxicity against K562 targets, assessed during the first treatment cycle for four E:T ratios. Data represents medians (and range) of % cytotoxicity

| | | *Cytotoxicity against K562 targets* | | | |
|---|---|---|---|---|---|
| | | *100:1* | *40:1* | *20:1* | *10:1* |
| Pretreatment cycle 1: | all pat. | 18.9 | 15.8 | 10.8 | 6.0 |
| | | (4.5–74.0) | (3.9–71.4) | (0.0–56.3) | (0.0–46.8) |
| Peak cycle 1: | all pat. | 50.0 | 40.4 | 26.7 | 18.5 |
| | | (28.0–74.0) | (15.4–71.4) | (9.6–56.3) | (7.6–52.3) |
| *P* (Wilcoxon test)[a] | | 0.002 | 0.002 | 0.002 | 0.002 |
| Pretreatment cycle 1: | CR/PR | 35.9 | 25.6 | 18.4 | 9.4 |
| | | (9.0–74.0) | (5.3–71.4) | (8.2–56.3) | (5.3–46.8) |
| | NC | 21.4 | 16.1 | 13.3 | 9.1 |
| | | (12.3–38.4) | (12.3–30.8) | (8.1–20.9) | (3.0–14.7) |
| | PD | 16.6 | 10.9 | 5.8 | 5.9 |
| | | (4.5–45.5) | (3.9–45.5) | (0.0–33.7) | (0.0–23.5) |
| *P* (Kruskal-Wallis test)[b] | | n.s. | n.s. | n.s. | n.s. |
| Peak cycle 1: | CR/PR | 58.8 | 48.5 | 36.3 | 27.9 |
| | | (43.0–74.0) | (27.4–71.4) | (19.4–56.3) | (10.2–52.3) |
| | NC | 40.8 | 33.3 | 22.6 | 16.5 |
| | | (26.4–73.9) | (20.8–69.0) | (16.7–41.8) | (13.9–32.3) |
| | PD | 50.9 | 40.0 | 25.2 | 19.7 |
| | | (33.7–64.4) | (15.4–64.4) | (9.6–50.2) | (7.6–32.2) |
| *P* (Kruskal-Wallis test)[b] | | n.s. | n.s. | n.s. | n.s. |

[a]Pretreatment *vs* peak. [b]CR/PR *vs* NC *vs* PD. n.s. = not significant.

**Table II** Pretreatment and peak cytotoxicity against Daudi targets, assessed during the first treatment cycle for four E:T ratios. Data represents medians (and range) of % cytotoxicity

| | | *Cytotoxicity against Daudi targets* | | | |
|---|---|---|---|---|---|
| | | *100:1* | *40:1* | *20:1* | *10:1* |
| Pretreatment cycle 1: | all pat. | 5.8 | 6.2 | 4.9 | 1.1 |
| | | (0.0–12.7) | (0.0–8.0) | (0.0–8.6) | (0.0–6.3) |
| Peak cycle 1: | all pat. | 34.8 | 30.0 | 21.2 | 15.0 |
| | | (7.4–57.7) | (7.1–56.8) | (7.3–35.7) | (2.3–27.5) |
| *P* (Wilcoxon test)[a] | | 0.001 | 0.002 | 0.001 | 0.001 |
| Pretreatment cycle 1: | CR/PR | 12.3 | 6.9 | 5.8 | 0.4 |
| | | (9.4–12.7) | (5.2–8.0) | (4.9–6.0) | (0.0–5.1) |
| | NC | 6.8 | 6.6 | 5.3 | 2.5 |
| | | (1.9–11.1) | (5.9–6.7) | (2.8–8.6) | (0.3–5.1) |
| | PD | 2.8 | 2.8 | 2.6 | 1.1 |
| | | (0.0–10.8) | (0.0–7.5) | (0.0–8.3) | (0.0–6.3) |
| *P* (Kruskal-Wallis test)[b] | | 0.036 | n.s. | n.s. | n.s. |
| *P* (Mann-Whitney test)[c] | | 0.030 | n.s. | n.s. | n.s. |
| Peak cycle 1: | CR/PR | 57.7 | 44.0 | 25.4 | 26.9 |
| | | (49.0–57.7) | (32.9–56.8) | (19.7–35.7) | (16.5–27.1) |
| | NC | 33.3 | 24.9 | 20.7 | 14.8 |
| | | (21.6–42.6) | (16.2–30.0) | (7.8–21.4) | (2.3–16.5) |
| | PD | 31.7 | 27.2 | 21.4 | 13.5 |
| | | (7.4–52.6) | (7.1–48.7) | (7.3–32.2) | (6.1–27.5) |
| *P* (Kruskal-Wallis test)[b] | | 0.049 | n.s. | n.s. | n.s. |
| *P* (Mann-Whitney test)[c] | | 0.025 | n.s. | n.s. | n.s. |

[a]Pretreatment *vs* peak. [b]CR/PR *vs* NC *vs* PD. [c]CR/PR *vs* PD. n.s. = not significant.

**Figure 2** **a** and **b**, Cytotoxicity against K562 targets (top) and against Daudi targets (bottom) during the first treatment cycle as assessed with four different E:T ratios. Data points represent median cytotoxicity for all patients. Shaded area = treatment period. -■- 100:1; -●- 40:1; -▲- 20:1; -◆- 10:1.

their clinical response to therapy, i.e. PR/CR *vs* NC *vs* PD. With both targets, median pretreatment as well as peak activities during the first cycle were greater in patients who subsequently achieved a PR/CR than in non-responders (Figures 3 and 4, Tables I and II). The differences between patient groups were statistically significant for the anti-Daudi activity at the E:T ratio of 100:1 but not at the lower ratios, and neither for the anti-K562 activity (Tables I and II). During the rest period after the first treatment course, responders were able to maintain higher cytotoxicity levels than non-responders.

*Cytotoxicity during subsequent treatment cycles* During the second treatment cycle, cytotoxicity levels were again related to the clinical response (Figures 3 and 4). Patients subsequently achieving a PR/CR generated higher activities against both targets than those with NC, and no significant augmentation of cytotoxicity was seen in patients with PD. No patient with PD received more than two treatment courses. Patients with NC were not able to maintain their cytotoxicity levels after the second course, in contrast to responders who preserved their high levels during periods without therapy.

*Surface markers: Leu-19 (CD 56)*

*Leu-19 expression during the first treatment cycle* The median percentages of peripheral blood Leu-19$^+$ cells as assessed by serial measurements before and during the first treatment cycle are depicted in Figure 5. In pretreatment samples, PBL positive for Leu-19 ranged from 4% to 20% (median 6%). Leu-19 expression increased continuously during treatment for a maximum on day 19 (median 24%, range 13–30%). As compared to day 1 (pretreatment), the relative number of Leu-19$^+$ cells was significantly augmented on days 12 and 19 ($P < 0.05$, Wilcoxon test).

**Figure 3** Cytotoxicity against K562 targets for patients with partial or complete response (PR/CR), no change (NC) of tumour parameters, and progressive disease (PD). Data points represent median cytotoxicity at an effector:target ratio of 100:1. Shaded areas = treatment periods (4 cycles).

**Figure 4** Cytotoxicity against Daudi targets for patients with partial or complete response (PR/CR), no change (NC) of tumour parameters, and progressive disease (PD). Data points represent median cytotoxicity at an effector:target ratio of 100:1. Shaded areas = treatment periods (4 cycles).

**Figure 5** Peripheral blood cells with positive staining for Leu-19 (CD 56) and IL-2 receptor (CD 25) during the first treatment cycle. Data points represent medians for 9 patients. Shaded area = treatment period.

*Relationship between Leu-19⁺ PBL and response* In pretreatment samples, no statistically significant differences in the percentage of Leu-19⁺ cells were found between responding *vs* non-responding patients (data not shown). During the first treatment cycle, peak percentages of Leu-19⁺ PBL in individual patients ranged from 15% to 32% (see Table III). The respective medians were 24% for all patients, 30% for patients achieving a CR/PR, and 20% for patients with PD. This difference between responders and non-responders reached statistical borderline significance (Table III).

### *Surface markers: IL-2 receptor (CD 25, Tac)*

*IL-2 receptor expression during the first treatment cycle* The proportion of CD 25⁺ peripheral blood cells was small in pretreatment samples (median 3%, range 1–13%). It can be seen from Figure 5 that this percentage increased during the first treatment cycle until day 12 (median 11%, range 5–28%), followed by a plateau phase through to day 19. Statistically significant increases since day 1 (pretreatment) were present on days 12 and 19 ($P<0.05$, Wilcoxon test).

*Relationship between IL-2 receptor expression and response* In pretreatment samples, relative numbers of CD 25⁺ peripheral blood cells were not significantly different between response groups (data not shown). During the first treatment course, individual patients achieved peak percentages of CD 25⁺ cells ranging from 5% to 28%, with medians of 12% for all patients, 9% for patients with subsequent CR/PR, and 14% for patients with PD, respectively (see Table III). This association between clinical remission and lower levels of CD 25⁺ cells was not statistically significant.

### *Proliferation assay*

*Spontaneous proliferation of PBL during* in vivo *treatment with IL-2/α-IFN* A certain variability in spontaneous PBL proliferation was present between individual patients. Nevertheless, a uniform treatment-related proliferation pattern was observed during the four therapy cycles. Figure 6 shows that the *in vivo* therapy always resulted in a temporary decrease of $^3$H-thymidine uptake, although this effect was minimal during the fourth cycle. Spontaneous proliferation recovered either later during treatment or during the treatment free interval. No significant correlation between clinical response and spontaneous proliferation was present (data not shown).

In vitro *stimulation of PBL with PHA* Before initiation of *in vivo* therapy, PHA at the two higher test concentrations induced proliferation of patients' PBL, whereas the lowest concentration (0.01 μg ml$^{-1}$) hardly had any effect. This is illustrated by median pretreatment SI of 88, 19, and 1.1, respectively. During *in vivo* therapy, the ability of PBL to proliferate in response to PHA showed no reproducible treatment-related pattern (see Figures 7a-c). SI showed high

**Figure 6** Spontaneous PBL proliferation (in medium alone). Data points represent medians of c.p.m. for all patients. Shaded areas = treatment periods (4 cycles).

**Figure 7** **a–c**, PBL proliferation after *in vitro* stimulation with PHA in three different concentrations for 96 h. Data points represent medians of SI for all patients. Shaded areas = treatment periods (4 cycles).

**Table III** Pretreatment and peak percentage of PBL positive for Leu-19 (CD 56) and IL-2 receptor (CD 25) during the first treatment cycle. Data represent medians (and range) for nine patients

| | | *Leu-19* | *IL-2 receptor* |
|---|---|---|---|
| Pretreatment cycle 1: | all pat. ($n = 9$) | 6.45 | 3.2 |
| | | (3.6–19.7) | (0.9–13.2) |
| Peak cycle 1: | all pat. ($n = 9$) | 24.1 | 11.7 |
| | | (15.1–32.2) | (5.4–28.3) |
| | $P$ (Wilcoxon test)[a] | 0.008 | 0.008 |
| Peak cycle 1: | CR/PR ($n = 2$) | 30.0 | 9.0 |
| | | (27.9–32.2) | (8.9–9.0) |
| | NC ($n = 1$) | 27.0 | 13.2 |
| | PD ($n = 6$) | 20.2 | 14.3 |
| | | (15.1–24.7) | (5.4–28.3) |
| | $P$ (Kruskal-Wallis test)[b] | 0.061 | n.s. |
| | $P$ (Mann-Whitney test)[c] | 0.046 | n.s. |

[a]Pretreatment *vs* peak. [b]Peak cycle 1, CR/PR *vs* NC *vs* PD. [c]Peak cycle 1, CR/PR *vs* PD. n.s. = not significant.

**Figure 8** PBL proliferation after *in vitro* stimulation with IL-2 in three different concentrations for 96 h. Data points represent medians of SI for all patients. Shaded areas = treatment periods (4 cycles).

interindividual (on a given sampling day) and intraindividual (during the whole study duration) variabilities. Proliferation of PBL in response to PHA was not associated with clinical response (data not shown).

In vitro *stimulation of PBL with IL-2* In pretreatment samples, IL-2 was capable of inducing PBL proliferation in a dose-dependent manner. Median SI for the three IL-2 concentrations tested were 11, 5, and 4, respectively (see Table IV). Proliferative responses of PBL to IL-2 temporarily increased during each treatment cycle (see Figure 8). The results for the first cycle are given in Table IV, and it can be seen that the differences beween pretreatment and peak SI were highly significant ($P \leqslant 0.005$ for each of the IL-2 concentrations, Wilcoxon test). Proliferative response of PBL to IL-2 was not associated with clinical responses (data not shown).

### Discussion

The present study demonstrates that concurrent administration of IL-2 and α-IFN to patients with advanced malignancy is biologically active. Our treatment regimen resulted in remarakable immunomodulation, most notably *in vivo* activation of PBL with induction of cytotoxicity against the two targets K562 and Daudi. K562 cells are very sensitive to NK cytolysis but may also be killed by LAK cells (Ortaldo & Longo, 1988). In contrast, Daudi cells are LAK-susceptible but relatively NK-resistant targets. It may therefore be concluded that the cytotoxicity induced by IL2/α-IFN treatment is largely due to *in vivo* generation of LAK activity. Augmented cytotoxicity levels were usually seen after the first five treatment days, but both anti-K562 and anti-Daudi activity continued to increase thereafter, and patients were capable of maintaining elevated cytotoxicity levels throughout the treatment period.

Moreover, levels of anti-K562 and anti-Daudi activity were found to be higher in patients who subsequently responded

**Table IV** Pretreatment and peak stimulation index (SI) during the first treatment cycle for stimulation of PBL with IL-2 in three concentrations. Data represent medians (and range) of SI for all patients

| | *IL-2* 1200 $IU\,ml^{-1}$ | 240 $IU\,ml^{-1}$ | 120 $IU\,ml^{-1}$ |
|---|---|---|---|
| Pretreatment cycle 1: | 10.6 | 5.1 | 4.0 |
| | (4.5–44.7) | (2.4–16.9) | (2.0–13.2) |
| Peak cycle 1: | 39.6 | 17.3 | 10.6 |
| | (8.9–90.9) | (4.9–64.7) | (3.9–60.6) |
| *P* (Wilcoxon test)[a] | 0.005 | 0.003 | 0.005 |

[a]Pretreatment *vs* peak.

to treatment than in non-responders. This correlation with subsequent clinical outcome was seen in the early phase and even before initiation of treatment, and it reached statistical significance for pretreatment anti-Daudi activity and peak activity against the same target during the first treatment cycle. However, differences between response groups became even more evident during and after the second treatment cycle. Responders were not only capable of generating higher cytotoxicity levels during treatment periods but also of maintaining higher levels during treatment free intervals. These results support preclinical data which indicate that generation of cytotoxic immune effector cells, particularly LAK cells, is a crucial step in achieving tumour regression by IL-2-based immunotherapy (Mulé *et al.*, 1986).

Although the number of published therapeutic trials using combined IL-2 and α-IFN is increasing, only few data are reported on monitoring of *in vivo* cytotoxicity. *In vivo* induction of cytotoxic activity against NK- and LAK-sensitive targets, but no consistent relationship between antitumour effect and extent of cytotoxicity, has been observed in a phase I trial (Budd *et al.*, 1989). Others (Pichert *et al.*, 1991) have investigated the inducible cytotoxicity (with additional *in vitro* stimulation of patients' PBL by IL-2 after IL-2/α-IFN treatment) which must not be mistaken for *in vivo* cytotoxicity (Lamers *et al.*, 1991). In one clinical trial, IL-2 has been used in combination with interferon-beta which, like α-IFN, is a type I interferon. Analysis of *in vivo* cytotoxicity in this study demonstrated a remarkable similarity to our data in so far as maximum killing of NK- and LAK-sensitive target cells was higher in responders than in non-responding patients, the difference being statistically significant for the activity against LAK-sensitive targets (Krigel *et al.*, 1990).

In contrast, *in vivo* generation of cytotoxicity has been monitored in a number of therapeutic studies using IL-2 alone (Creekmore *et al.*, 1989; Gambacorti-Passerini *et al.*, 1988; Ghosh *et al.*, 1989; Klasa *et al.*, 1990; McMannis *et al.*, 1988; Paciucci *et al.*, 1989; Rosenthal *et al.*, 1988; Sondel *et al.*, 1988; Sosman *et al.*, 1988). Treatment-induced augmentation of activity against NK-sensitive targets was a common observation. In contrast, *in vivo* generation of LAK activity was not seen consistently. It appears from these studies that optimum *in vivo* LAK cell generation requires an IL-2 treatment for at least four consecutive days, corresponding to the *in vitro* LAK cell generation which requires a minimum duration of 3 days for culture of PBL in IL-2. Moreover, the schedule and route of IL-2 administration is an additional critical factor in obtaining optimum LAK cell activity. For instance, it has become clear that IL-2 given as a continuous intravenous (i.v.) infusion has greater biological effects than the same total dose given as bolus i.v. injection(s) (Kohler *et al.*, 1989; Thompson *et al.*, 1989). In order to get best *in vivo* induction of LAK activity, prolonged therapeutic IL-2 serum concentrations may therefore be superior to short though high peak levels. Pharmacokinetic studies suggest that a single IL-2 dose of $18 \times 10^6$ IU, as applied in our regimen, given subcutaneously to a typical human with a body surface area of 1.7 $m^2$ will result in a serum level of approximately 100 IU $ml^{-1}$ (Konrad *et al.*, 1990). This is only 2% of the level which is obtained if the same dose is given as an i.v. bolus, but it is in the same range as the steady state concentration which is obtained after continuous infusion of the same dose over 24 h. Moreover, the serum concentration after a subcutaneous injection remains fairly constant for about 8 h, whereas the serum level after bolus i.v. administration decreases with a half-life of 12.9 min (Konrad *et al.*, 1990). These data may explain the remarkably high *in vivo* cytotoxicity levels seen in our study although low to moderate (outpatient) drug dosages have been used. However, our study design was not suitable to assess the relative contribution of α-IFN to the process of cytotoxicity generation.

Most therapeutic studies using IL-2 alone, including one performed in our own institution (Ghosh *et al.*, 1989), failed to detect any significant differences in *in vivo* cytotoxicity between responding and non-responding patients. A few trials, however, reported on higher cytotoxicity levels in patients with clinical response (Klasa *et al.*, 1990; Paciucci *et al.*, 1989; Yasumoto *et al.*, 1987). The variability in these findings suggests that many factors related both to the treatment and to the cytotoxicity assay (frequency and timing of assays, expression of cytolytic activity as percentage cytotoxicity or as lytic units, target cells and E:T ratios used) may be crucial for detection of a significant correlation between cytotoxicity levels and clinical outcome.

Leu-19 is a surface marker which is expressed by a minority of normal PBL, and Leu-$19^+$ cells typically mediate NK cytotoxicity (Lanier *et al.*, 1986). There is some evidence that LAK cells generated *in vivo* are largely confined to the Leu-$19^+$ cell population although they are heterogeneous for co-expression of additional markers (Ellis *et al.*, 1988; McMannis *et al.*, 1988; Weil-Hillman *et al.*, 1989). In the present study we were able to demonstrate a statistically significant increase in the relative number of Leu-$19^+$ cells during IL-2/α-IFN treatment. This result is in agreement with findings in other clinical studies using IL-2 in combination with α-IFN (Budd *et al.*, 1990; Pichert *et al.*, 1991; Zinzani *et al.*, 1990) or alone (Creekmore *et al.*, 1989; Klasa *et al.*, 1990; Sosman *et al.*, 1988). The proportion of Leu-$19^+$ cells increased throughout the first treatment cyclie, the peak percengage being reached at the end of the 3 week treatment period. A continuing expansion of Leu-$19^+$ lymphocytes during the whole treatment period was also observed in a study where patients were treated with IL-2 alone for 4 weeks (Sosman *et al.*, 1988).

The CD 25 molecule (Tac antigen), which is expressed by activated lymphocytes and monocytes, is known to function as low affinity receptor for IL-2. There is clear evidence from *in vitro* studies that the proportion of CD $25^+$ cells increases after incubation of PBL with IL-2, but this process is partially inhibited when additional α-IFN is present in the cultures (Di Raimondo *et al.*, 1987; Sone *et al.*, 1988; Tokuda *et al.*, 1989). Increased percentages of CD $25^+$ lymphocytes have also been observed after *in vivo* treatment with IL-2 for several consecutive days (Gambacorti-Passerini *et al.*, 1988; Ghosh *et al.*, 1989; Klasa *et al.*, 1990; Sondel *et al.*, 1988; Sosman *et al.*, 1988) whilst regimens using a single weekly IL-2 dose had little if any effect (Atkins *et al.*, 1986; Creekmore *et al.*, 1989; Thompson *et al.*, 1987). In our study the relative number of CD $25^+$ peripheral lymphocytes was found to increase significantly after initiation of treatment for a peak on day 12, followed by a plateau phase through to day 19, thus indicating that maximum cell activation was achieved after the second treatment week. The similar kinetics of CD $25^+$ expression and cytotoxic activity during the first treatment cycle is noteworthy.

Interestingly the phenotype analysis in our study revealed that peak percentages of Leu-$19^+$ PBL were higher in responding patients than in patients with progressing disease. In contrast, peak percentages of CD $25^+$ cells were lower in responders than in non-responders. These results are remarkably consistent with data recently reported by another group (Kirchner *et al.*, 1990; Lopez Hänninen *et al.*, 1991). In their study, the same correlations between clinical response and expression of Leu-19 and CD 25 by peripheral blood cells were observed in cancer patients who were, as in our study, treated with concomitant subcutaneous IL-2 and α-IFN for a prolonged period.

A prominent effect of IL-2 is the induction of PBL proliferation (Bich-Thuy *et al.*, 1986). In contrast, α-IFN has antiproliferative properties, and proliferation of human PBL (as well as murine spleen cells) is inhibited after co-incubation with IL-2 and α-IFN as compared to culture in IL-2 alone (Brunda *et al.*, 1986; Di Raimondo *et al.*, 1987; Sone *et al.*, 1988; Tokuda *et al.*, 1989). In our study, patients' PBL obtained repeatedly during *in vivo* treatment were tested for their ability to proliferate *in vitro* by incubation in medium alone, i.e. without further *in vitro* drug exposure. This spontaneous PBL proliferation was clearly inhibited by the *in vivo* treatment, a result which is in agreement with the *in vitro* data mentioned. In contrast, augmented spontaneous PBL proliferation was observed after *in vivo* treatment with IL-2 alone (Gambacorti-Passerini *et al.*, 1988). It is therefore most likely that the inhibition of PBL proliferation in our study was due to the antiproliferative effect of α-IFN. This conclusion is supported by the observation that spontaneous PBL proliferation recovered during the treatment free intervals.

There is some evidence that generation of LAK cytotoxicity results from a functional activation rather than from a proliferative expansion of LAK precursors (Blay *et al.*, 1989; Ramsdell *et al.*, 1988). Moreover, kinetic studies revealed that IL-2 induced enhancement of NK cytotoxicity in PBL preceded any proliferation (Trinchieri *et al.*, 1984). Therefore induction of cytotoxic activity and lymphocyte proliferation seem to be two largely independent processes following IL-2 exposure which do not necessarily need to be concordant, and the relatively high cytotoxicity levels observed in our study are not in contradiction to the inhibition of PBL proliferation.

We have also serially examined the proliferative responses of patients' PBL to *in vitro* stimulation with IL-2 and PHA. Augmented PBL proliferation in the presence of IL-2 *in vitro* was consistently seen during all four *in vivo* treatment cycles. This finding indicates that the *in vivo* treatment resulted in activation of a PBL subpopulation which was capable of responding with more rapid kinetics to IL-2 *in vitro*. Similar results were seen in patients who have been treated with IL-2 alone over several consecutive days (Rosenthal *et al.*, 1988; Sosman *et al.*, 1988). In contrast, PBL proliferation in response to PHA, a T cell mitogen, did not appear to change reproducibly during treatment, and the variability between individual patients was high. Comparable observations were reported when patients' PBL were stimulated with PHA *in vitro* after *in vivo* treatment with IL-2 alone (Paciucci *et al.*, 1989; Rosenthal *et al.*, 1988; Sosman *et al.*, 1988; Thompson *et al.*, 1987).

In conclusion, remarkable immunomodulatory effects were seen in the present study. Our data have been generated by a relatively small number of patients and need to be confirmed in larger patient series. The significant correlation between *in vivo* cytotoxicity against Daudi targets and response to therapy is of special clinical interest, since immune parameters correlating with subsequent clinical outcome in the early phase of immunotherapy might be helpful in selecting patients who are likely to respond to prolonged treatment. This issue should be addressed by future studies.

A. von Rohr is in receipt of a fellowship provided by the European Society for Medical Oncology (ESMO).

### References

ATKINS, M.B., GOULD, J.A., ALLEGRETTA, M., LI, J.J., DEMPSEY, R.A., RUDDERS, R.A., PARKINSON, D.R., REICHLIN, S. & MIER, J.W. (1986). Phase I evaluation of recombinant interleukin-2 in patients with advanced malignant disease. *J. Clin. Oncol.*, **4**, 1380–1391.

BICH-THUY, L.T., LANE, H.C. & FAUCI, A.S. (1986). Recombinant interleukin-2-induced polyclonal proliferation of *in vitro* unstimulated human peripheral blood lymphocytes. *Cell. Immunol.*, **98**, 396–410.

BLAY, J.Y., BERTOGLIO, J., FRADELIZI, D. & CHOUAIB, S. (1989). Functional interactions of IL2 and TNF in the differentiation of LGL into LAK effectors. *Int. J. Cancer*, **44**, 598–604.

BRUNDA, M.J., BELLANTONI, D. & SULICH, V. (1987). *In vivo* antitumor activity of combinations of interferon alpha and interleukin-2 in a murine model. Correlation of efficacy with the induction of cytotoxic cells resembling natural killer cells. *Int. J. Cancer*, **40**, 365–371.

BRUNDA, M.J. & DAVATELIS, V. (1985). Augmentation of natural killer cell activity by recombinant interleukin-2 and recombinant interferons. In *Mechanisms of Cytotoxicity by NK Cells*, Herberman, R.B. & Callewaert, D.M. (eds), pp. 397–407. Academic Press: Orlando/San Diego/New York/London/Toronto/Montreal/Sydney/Tokyo.

BRUNDA, M.J., TARNOWSKI, D. & DAVATELIS, V. (1986). Interaction of recombinant interferons with recombinant interleukin-2: differential effects on natural killer cell activity and interleukin-2-activated killer cells. *Int. J. Cancer*, **37**, 787–793.

BUDD, G.T., OSGOOD, B., BARNA, B., BOYETT, J.M., FINKE, J., MEDENDORP, S.V., MURTHY, S., NOVAK, C., SERGI, J., TUBBS, R. & BUKOWSKI, R.M. (1989). Phase I clinical trial of interleukin 2 and α-interferon: toxicity and immunologic effects. *Cancer Res.*, **49**, 6432–6436.

BUDD, G.T., SERGI, J., FINKE, J., TUBBS, R., MURTHY, S., GIBSON, V., MEDENDORP, S., BAUER, L., BARNA, B., BOYETT, J. & BUKOWSKI, R.M. (1990). Combination interleukin-2 and alpha-interferon therapy of metastatic renal cell carcinoma and malignant melanoma. *AACR Proc.*, **31**, 271 (#1603).

CAMERON, R.B., MCINTOSH, J.K. & ROSENBERG, S.A. (1988). Synergistic antitumor effects of combination immunotherapy with recombinant interleukin-2 and a recombinant hybrid α-interferon in the treatment of established murine hepatic metastases. *Cancer Res.*, **48**, 5810–5817.

CHIKKALA, N.F., LEWIS, I., ULCHAKER, J., STANLEY, J., TUBBS, R. & J.H., F. (1990) Interactive effects of α-Interferon A/D and interleukin 2 on murine lymphokine-activated killer activity: analysis at the effector and precursor level. *Cancer Res.*, **50**, 1176–1182.

CREEKMORE, S.P., HARRIS, J.E., ELLIS, T.M., BRAUN, D.P., COHEN, I.I., BHOOPALAM, N., JASSAK, P.F., CAHILL, M.A., CANZONERI, C.L. & FISHER, R.I. (1989). A phase I clinical trial of recombinant interleukin-2 by periodic 24-hour intravenous infusions. *J. Clin. Oncol.*, **7**, 276–284.

CZARNIECKI, C.W., FENNIE, C.W., POWERS, D.B. & ESTELL, D.A. (1984). Synergistic antiviral and antiproliferative activities of *Escherichia coli*-derived human alpha, beta and gamma interferons. *J. Virol.*, **49**, 490–496.

DI RAIMONDO, F., LAPUSHIN, R. & HERSH, E.M. (1987). Synergism between alpha-interferon and interleukin-2-activated killer cells: in vitro studies. *Acta Haemat.*, **78** (Suppl. 1), 77–83.

ELLIS, T.M., CREEKMORE, S.P., MCMANNIS, J.D., D.P., B., HARRIS, J.A. & FISHER, R.I. (1988). Appearance and phenotypic characterization of circulating Leu 19+ cells in cancer patients receiving recombinant interleukin 2. *Cancer Res.*, **48**, 6597–6602.

FIDLER, I.J., HEICAPPELL, R., SAIKI, I., GRUTTER, M.G., HORISBERGER, M.A. & NUESCH, J. (1987). Direct antiproliferative effects of recombinant human interferon-α- B/D hybrids on human tumor cell lines. *Cancer Res.*, **47**, 2020–2027.

GAMBACORTI-PASSERINI, C., RADRIZZANI, M., MAROLDA, R., BELLI, F., SCIORELLI, G., GALAZKA, A.R., SCHINDLER, J.D., CASCINELLI, N. & PARMIANI, G. (1988). *In vivo* activation of lymphocytes in melanoma patients receiving escalating doses of recombinant interleukin 2. *Int. J. Cancer*, **41**, 700–706.

GHOSH, A.K., DAZZI, H., THATCHER, N. & MOORE, M. (1989). Lack of correlation between peripheral blood lymphokine-activated killer (LAK) cell function and clinical response in patients with advanced malignant melanoma receiving recombinant interleukin 2. *Int. J. Cancer*, **43**, 410–414.

GIACOMINI, P., AGUZZI, A., PESTKA, S., FISHER, P.B. & FERRONE, S. (1984). Modulation by recombinant DNA leucocyte (α) and fibroblast (β) interferons of the expression and shedding of HLA- and tumor-associated antigens by human melanoma cells. *J. Immunol.*, **133**, 1649–1655.

GREENBERG, A.H., MILLER, V., JABLONSKI, T. & POHAJDAK, B. (1984). Suppression of NK-mediated natural resistance by interferon treatment of murine lymphomas. *J. Immunol.*, **132**, 2129–2134.

GRIMM, E.A., MAZUMDER, A., ZHANG, H.Z. & ROSENBERG, S.A. (1982). Lymphokine-activated killer cell phenomenon: lysis of natural killer-resistant fresh solid tumor cells by interleukin 2-activated autologous human peripheral blood lymphocytes. *J. Exp. Med.*, **155**, 1823–1841.

HENNEY, C.S., KURIBAYASHI, K., KERN, D.E. & GILLIS, S. (1981). Interleukin-2 augments natural killer cell activity. *Nature*, **291**, 335–338.

HERBERMAN, R.B., ORTALDO, J.R., MANTOVANI, A., HOBBS, D.S., KUNG, H.F. & PESTKA, S. (1982). Effect of human recombinant interferon on cytotoxic activity of natural killer (NK) cells and monocytes. *Cell. Immunol.*, **67**, 160–167.

HICKS, N.J., MORRIS, A.G. & BURKE, D.C. (1981). Partial reversion of the transformed phenotype of murine sarcoma virus-transformed cells in the presence of interferon: a possible mechanism for the anti-tumour effect of interferon. *J. Cell Sci.*, **49**, 225–236.

IIGO, M., NAKAJIMA, Y., NISHIKATA, K. & HOSHI, A. (1989). Effects of interleukin-2 and interferon-αA/D treatment on lymphocytes from tumour-bearing mice. *Br. J. Cancer*, **59**, 883–888.

KIM, B., WARNAKA, P. & IMBEMBO, A. (1989). Interleukin-2 and alpha interferon therapy of advanced pulmonary metastases. *Eur. Surg. Res.*, **21**, 260–266.

KIRCHNER, H., KÖRFER, A., PALMER, P.A., EVERS, P., DE RIESE, W., KNÜVER-HOPF, J., HADAM, M., GOLDMANN, U., FRANKS, C.R., POLIWODA, H. & ATZPODIEN, J. (1990). Subcutaneous interleukin-2 and interferon-α2b in patients with metastatic renal cell cancer: the German outpatient experience. *Mol. Biother.*, **2**, 145–154.

KLASA, R.J., SILVER, H.K.B. & KONG, S. (1990). *In vivo* induction of lymphokine-activated killer cells by interleukin-2 splenic artery perfusion in advanced malignancy. *Cancer Res.*, **50**, 4906–4910.

KOHLER, P.C., HANK, J.A., MOORE, K.H., STORER, B., BECHHOFER, R., HONG, R. & SONDEL, P.M. (1989). Phase I clinical trial of recombinant interleukin-2: a comparison of bolus and continuous intravenous infusion. *Cancer Invest.*, **7**, 213–223.

KONRAD, M.W., HEMSTREET, G., HERSH, E.M., MANSELL, P.W.A., MERTELSMANN, R., KOLITZ, J.E. & BRADLEY, E.C. (1990). Pharmacokinetics of recombinant interleukin 2 in humans. *Cancer Res.*, **50**, 2009–2017.

KRIGEL, R.L., PADAVIC-SHALLER, K.A., RUDOLPH, A.R., KONRAD, M., BRADLEY, E.C. & COMIS, R.L. (1990). Renal cell carcinoma: treatment with recombinant interleukin-2 plus beta-interferon. *J. Clin. Oncol.*, **8**, 460–467.

LAMERS, C.H.J., GRATAMA, J.W., VAN PUTTEN, W.L.J., STOTER, G. & BOLHUIS, R.L.H. (1991). Exogenous interleukin 2 recruits *in vitro* lymphokine-activated killer activity by *in vivo* activated lymphocytes. *Cancer Res.*, **51**, 2324–2328.

LANIER, L.L., MY LE, A., CIVIN, C.I., LOKEN, M.R. & PHILLIPS, J.H. (1986). The relationship of CD16 (Leu-11) and Leu-19 (NKH-1) antigen expression on human peripheral blood NK cells and cytotoxic T lymphocytes. *J. Immunol.*, **136**, 4480–4485.

LOPEZ HÄNNINEN, E., KÖRFER, A., HADAM, M., SCHNEEKLOTH, C., DALLMANN, I., MENZEL, T., KIRCHNER, H., POLIWODA, H. & ATZPODIEN, J. (1991). Biological monitoring of low-dose interleukin 2 in humans: soluble interleukin 2 receptors, cytokines, and cell surface phenotypes. *Cancer Res.*, **50**, 6312–6316.

LOTZE, M.T., CUSTER, M.C., SHARROW, S.O., RUBIN, L.A., NELSON, D.L. & ROSENBERG, S.A. (1987). *In vivo* administration of purified human interleukin-2 to patients with cancer: development of interleukin-2 receptor positive cells and circulating soluble interleukin-2 receptors following interleukin-2 administration. *Cancer Res.*, **47**, 2188–2195.

MCMANNIS, J.D., FISHER, R.I., CREEKMORE, S.P., BRAUN, D.P., HARRIS, J.E. & ELLIS, T.M. (1988). *In vivo* effects of recombinant IL-2. I. Isolation of circulating Leu-19$^+$ lymphokine-activated killer effector cells from cancer patients receiving recombinant IL-2. *J. Immunol.*, **1988**, 1335–1340.

METHA, S., WEIGEL, T., ZHANG, S., TIBBETTS, L. & WANEBO, H. (1991). Differential cytokine effects on LAK cell lysis: induction of target cell resistance by interferon alpha (IFNα-. *ASCO Proc.*, **10**, 215 (#721).

MULÉ, J.J., YANG, J., SHU, S. & ROSENBERG, S.A. (1986). The antitumor efficacy of lymphokine-activated killer cells and recombinant interleukin 2 *in vivo*: direct correlation between reduction of established metastases and cytolytic activity of lymphokine-activated killer cells. *J. Immunol.*, **136**, 3899–3909.

ORTALDO, J.R. & LONGO, D.L. (1988). Human natural lymphocyte effector cells: definition, analysis of activity, and clinical effectiveness. *J. Natl Cancer Inst.*, **80**, 999–1010.

ORTALDO, J.R., MASON, A. & OVERTON, R. (1986). Lymphokine-activated killer cells: analysis of progenitors and effectors. *J. Exp. Med.*, **164**, 1193–1205.

PACIUCCI, P.A., HOLLAND, J.F., RYDER, J.S., KONEFAL, R.G., BEKESI, G.J., ODCHIMAR, R. & GORDON, R. (1989). Immunotherapy with interleukin-2 by constant infusion with and without adoptive cell transfer and with weekly doxorubicin. *Cancer Treat. Rev.*, **16** (Suppl. A), 67–81.

PHILLIPS, J.H. & LANIER, L.L. (1986). Dissection of the lymphokine-activated killer phenomenon. Relative contribution of peripheral blood natural killer cells and T-lymphocytes to cytolysis. *J. Exp. Med.*, **164**, 814–825.

PICHERT, G., JOST, L.M., FIERZ, W. & STAHEL, R.A. (1991). Clinical and immune modulatory effects of alternative weekly interleukin-2 and interferon alfa-2a in patients with advanced renal cell carcinoma and melanoma. *Br. J. Cancer*, **63**, 287–292.

RAMSDELL, F.J., SHAU, H. & GOLUB, S.H. (1988). Role of proliferation in LAK cell development. *Cancer Immunol. Immunother.*, **26**, 139–144.

ROBERTSON, M.J. & RITZ, J. (1990). Biology and clinical relevance of human natural killer cells. *Blood*, **76**, 2421–2438.

ROSENBERG, S.A., SCHWARZ, S.L. & SPIESS, P.J. (1988). Combination immunotherapy for cancer: synergistic antitumor interactions of interleukin-2, alfa interferon, and tumor-infiltrating lymphocytes. *J. Natl Cancer Inst.*, **80**, 1393–1397.

ROSENTHAL, N.S., HANK, J.A., KOHLER, P.C., MINKOFF, D.Z., MOORE, K.H., BECHHOFER,R., HONG, R., STORER, B. & SONDEL, P.M. (1988). The in vitro function of lymphocytes from 25 cancer patients receiving four to seven consecutive days of recombinant IL-2. *J. Biol. Response Mod.*, **7**, 123–139.

SONDEL, P.M., HANK, J.A., KOHLER, P.C., CHEN, P.B., MINKOFF, D.Z. & MOLENDA, J.A. (1986). Destruction of autologous human lymphocytes by interleukin-2 activated cytotoxic cells. *J. Immunol.*, **137**, 502–511.

SONDEL, P.M., KOHLER, P.C., HANK, J.A., MOORE, K.H., ROSENTHAL, N.S., SOSMAN, J.A., BECHHOFER, R.A. & STORER, B. (1988). Clinical and immunological effects of recombinaint interleukin 2 given by repetitive weekly cycles to patients with cancer. *Cancer Res.*, **48**, 2561–2567.

SONE, S., UTSUGI, T., NII, A. & OGURA, T. (1988). Differential effects of recombinant interferons α, β, and γ on induction of human lymphokine (IL-2)-activated killer activity. *J. Natl Cancer Inst.*, **80**, 425–431.

SOSMAN, J.A., KOHLER, P.C., HANK, J.A., MOORE, K.H., BECHHOFER, R., STORER, B. & SONDEL, P.M. (1988). Repetitive weekly cycles of interleukin-2. II. Clinical and immunologic effects of dose, schedule, and addition of indomethacin. *J. Natl Cancer Inst.*, **80**, 1451–1461.

THOMPSON, J.A., LEE, D.J., COX, W.W., LINDGREN, C.G., COLLINS, C., NERAAS, K.A., DENNIN, R.A. & FEFER, A. (1987). Recombinant interleukin 2 toxicity, pharmakokinetics, and immunomodulatory effects in a phase I trial. *Cancer Res.*, **47**, 4202–4207.

THOMPSON, J.A., LEE, D.J., LINDGREN, C.G., BENZ, L.A., COLLINS, C., SHUMAN, W.P., LEVITT, D. & FEFER, A. (1989). Influence of schedule of interleukin 2 administration on therapy with interleukin 2 and lymphokine activated killer cells. *Cancer Res.*, **49**, 235–240.

TOKUDA, Y., EBINA, N. & GOLUB, S.H. (1989). The inhibitory effect of human interferon α on the generation of lymphokine-activated killer activity. *Cancer Immunol. Immunother.*, **30**, 205–212.

TRINCHIERI, G., GRANATO, D. & PERUSSIA, B. (1981). Interferon-induced resistance of fibroblasts to cytolysis mediated by natural killer cells: specificity and mechanism. *J. Immunol.*, **126**, 335–340.

TRINCHIERI, G., MATSUMOTO-KOBAYASHI, M., CLARK, S.C., SEEHRA, J., LONDON, L. & PERUSSIA, B. (1984). Response of resting human peripheral blood natural killer cells to interleukin 2. *J. Exp. Med.*, **160**, 1147–1169.

WEBER, J.S. & ROSENBERG, S.A. (1988). Modulation of murine tumor major histocompatibility antigens by cytokines *in vivo* and *in vitro*. *Cancer Res.*, **48**, 5818–5824.

WEIL-HILLMAN, G., FISCH, P., PRIEVE, A.F., SOSMAN, J.A., HANK, J.A. & SONDEL, P.M. (1989). Lymphokine-activated killer cell activity induced by *in vivo* interleukin 2 therapy: predominant role for lymphocytes with increased expression of CD2 and Leu19 antigens but negative expression of CD16 antigens. *Cancer Res.*, **49**, 3680–3688.

YAMADA, S., RUSCETTI, F.W., OVERTON, W.R., HERBERMAN, R.B., BIRCHENALL-SPARKS, M.C. & ORTALDO, J.R. (1987). Regulation of human large granular lymphocyte and T cell growth and function by recombinant interleukin 2: induction of interleukin 2 receptor and promotion of growth of cells with enhanced cytotoxicity. *J. Leucocyte Biol.*, **41**, 505–517.

YASUMOTO, K., MIYAZAKI, K., NAGASHIMA, A., ISHIDA, T., KUDA, T., YANO, T., SUGIMACHI, K. & NOMOTO, K. (1987). Induction of lymphokine-activated killer cells by intrapleural instillations of recombinant interleukin-2 in patients with malignant pleurisy due to lung cancer. *Cancer Res.*, **47**, 2184–2187.

ZINZANI, P.L., CLARK, J.W., WANEBO, H.J., KOUTTAB, N.M. & CALABRESI, P. (1990). Continuous intravenous infusion interleukin-2 (IL-2) and α-interferon (α-IFN) therapy for melanoma with *in vitro* studies of combination cytokines for melanoma. *ASCO Proc.*, **9**, 279 (#1081).


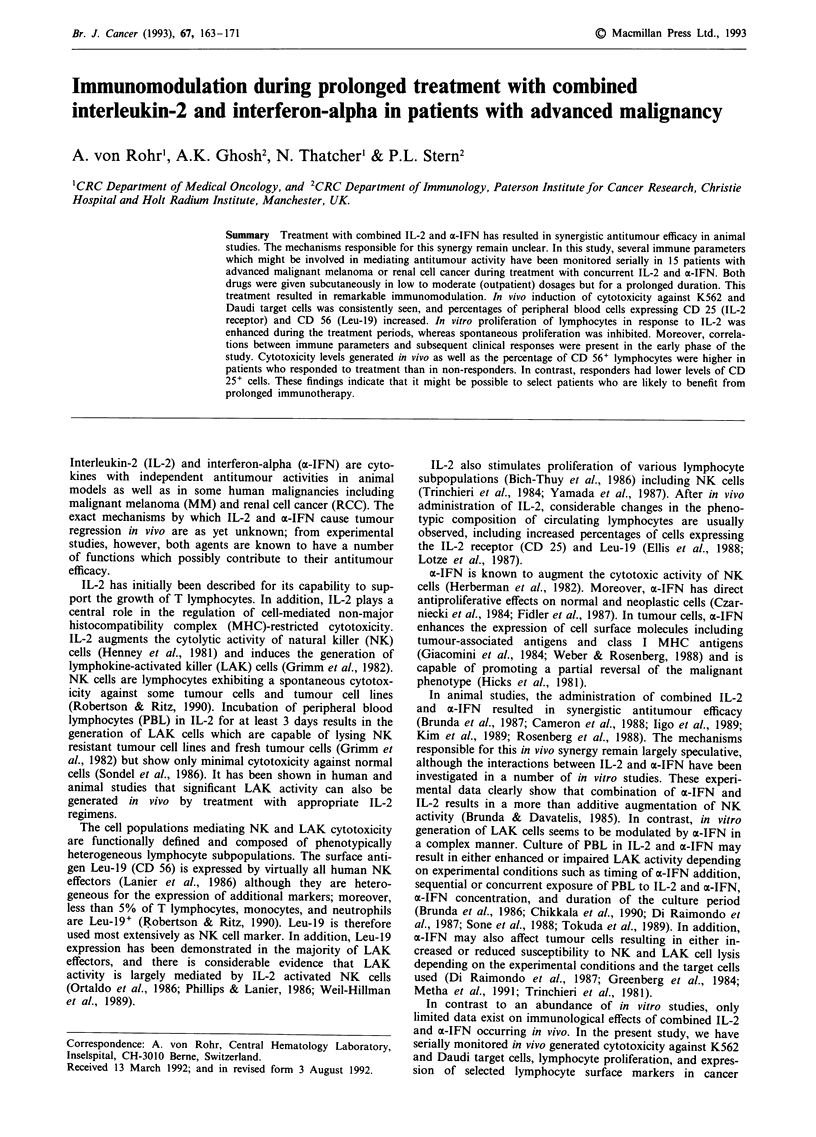

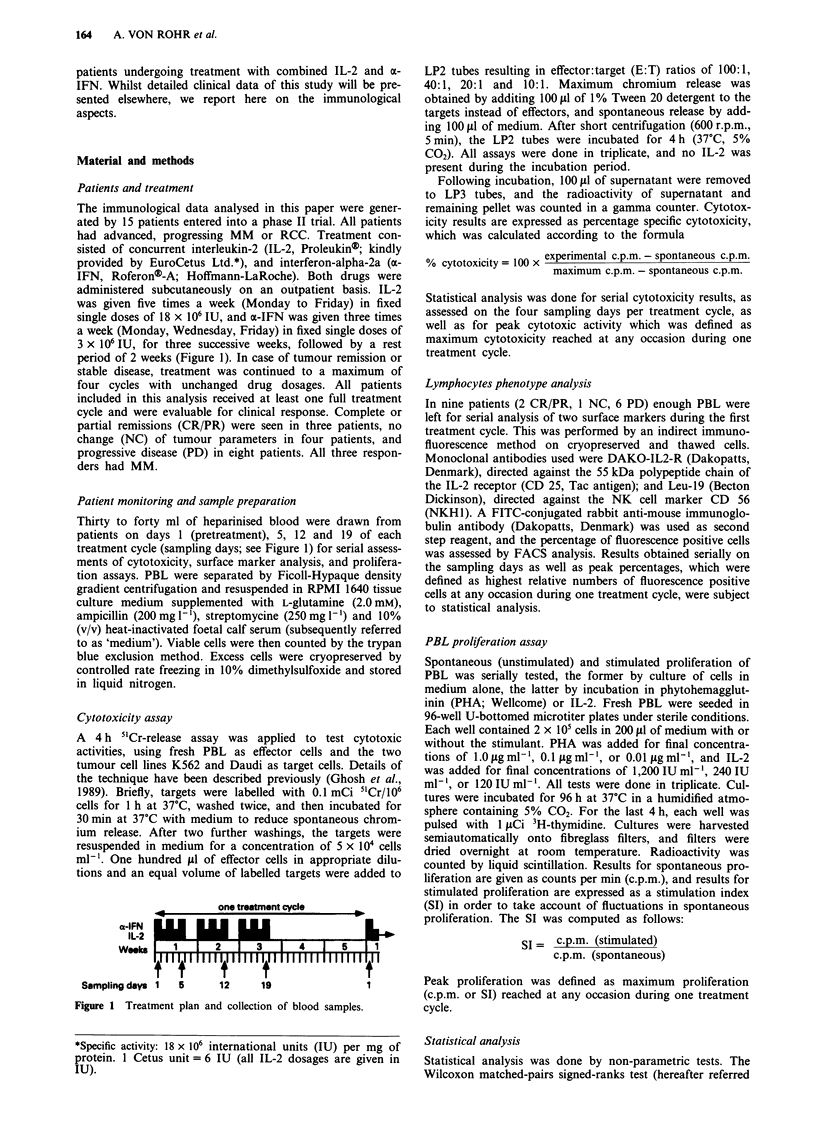

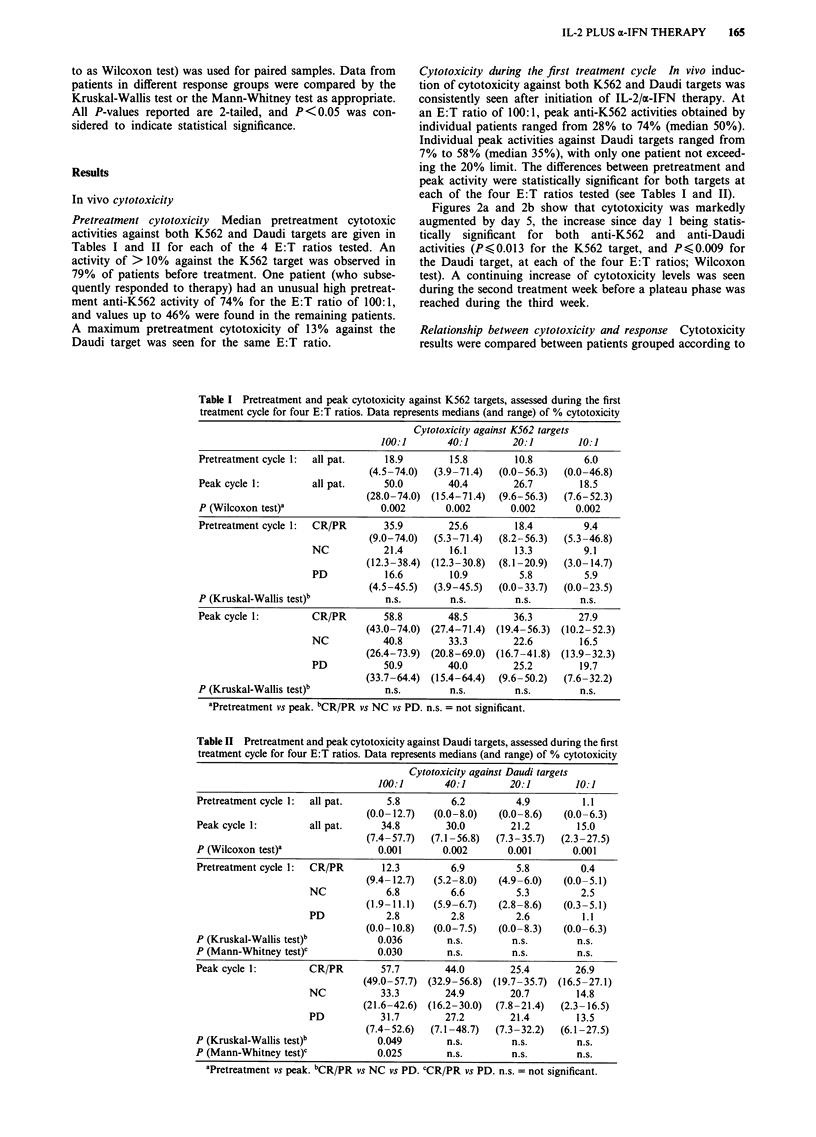

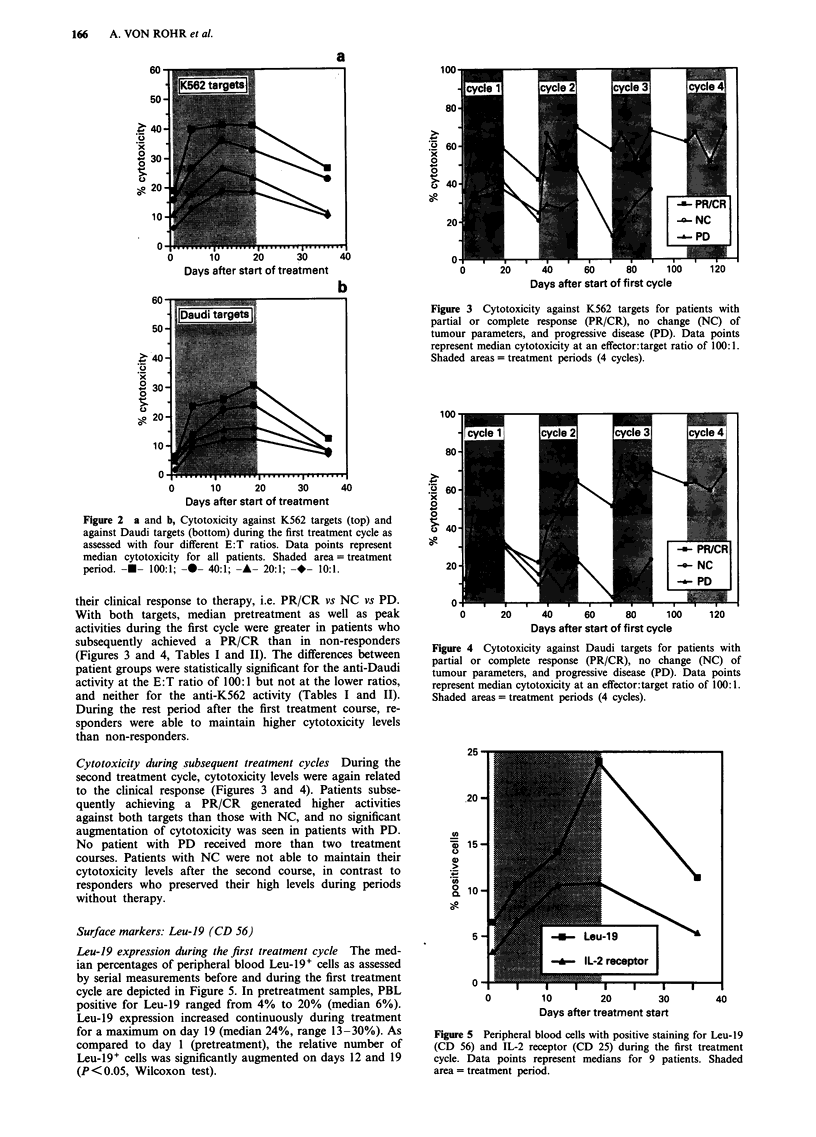

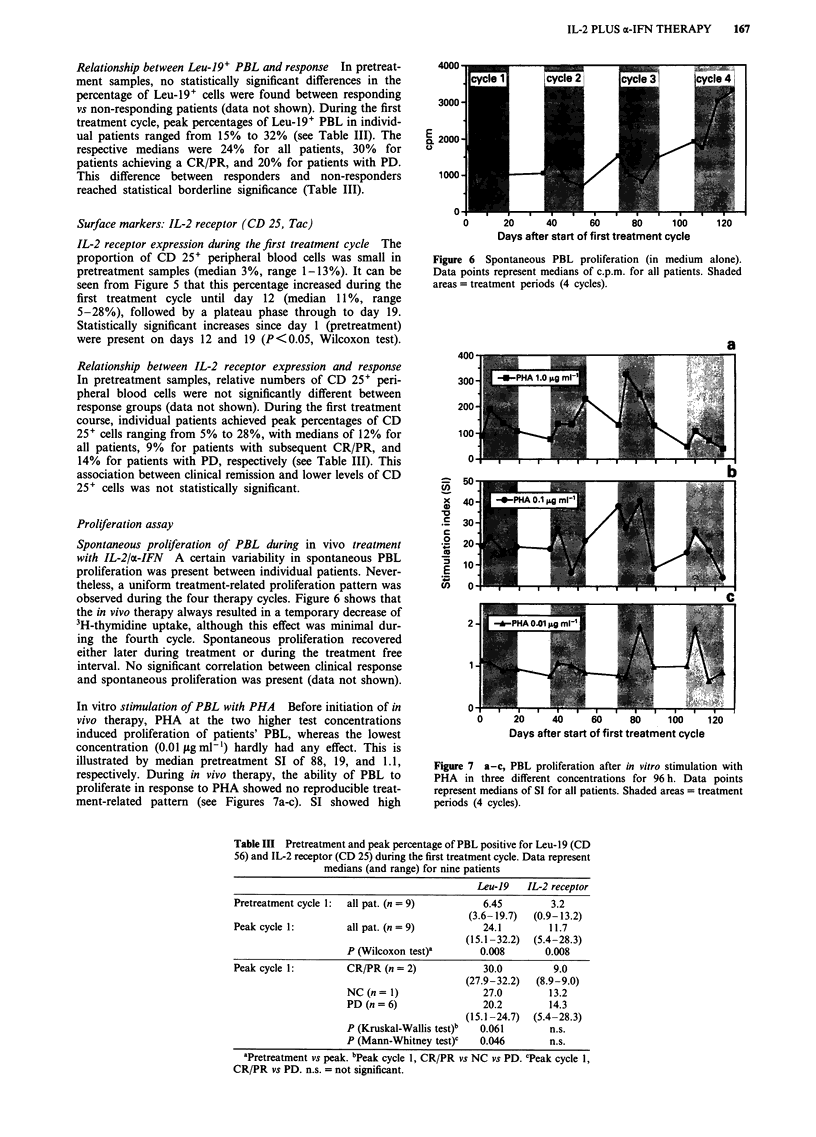

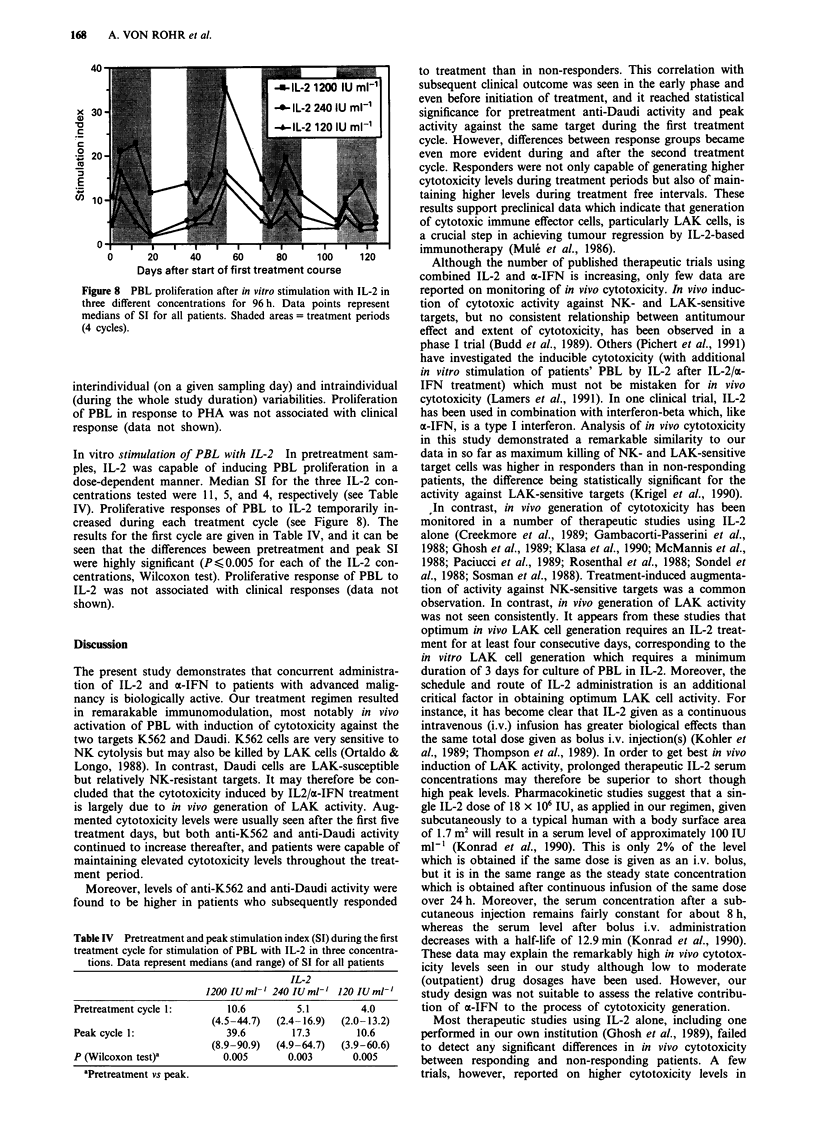

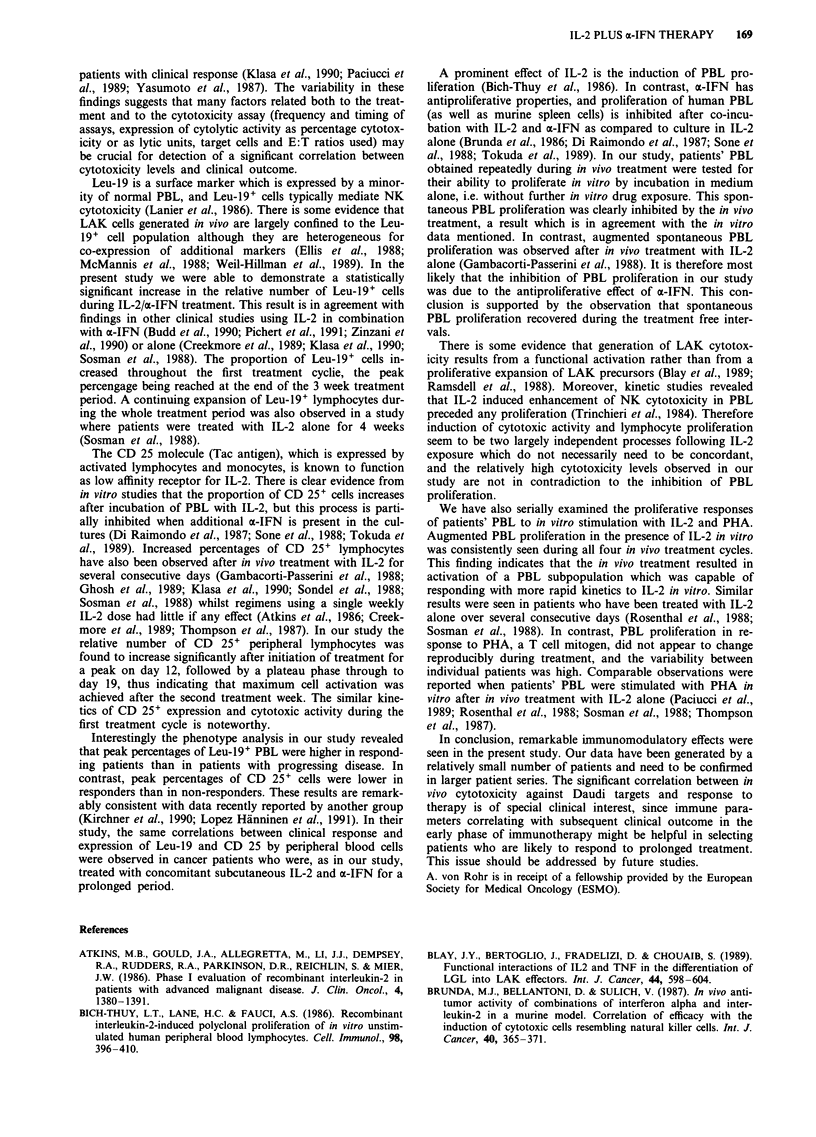

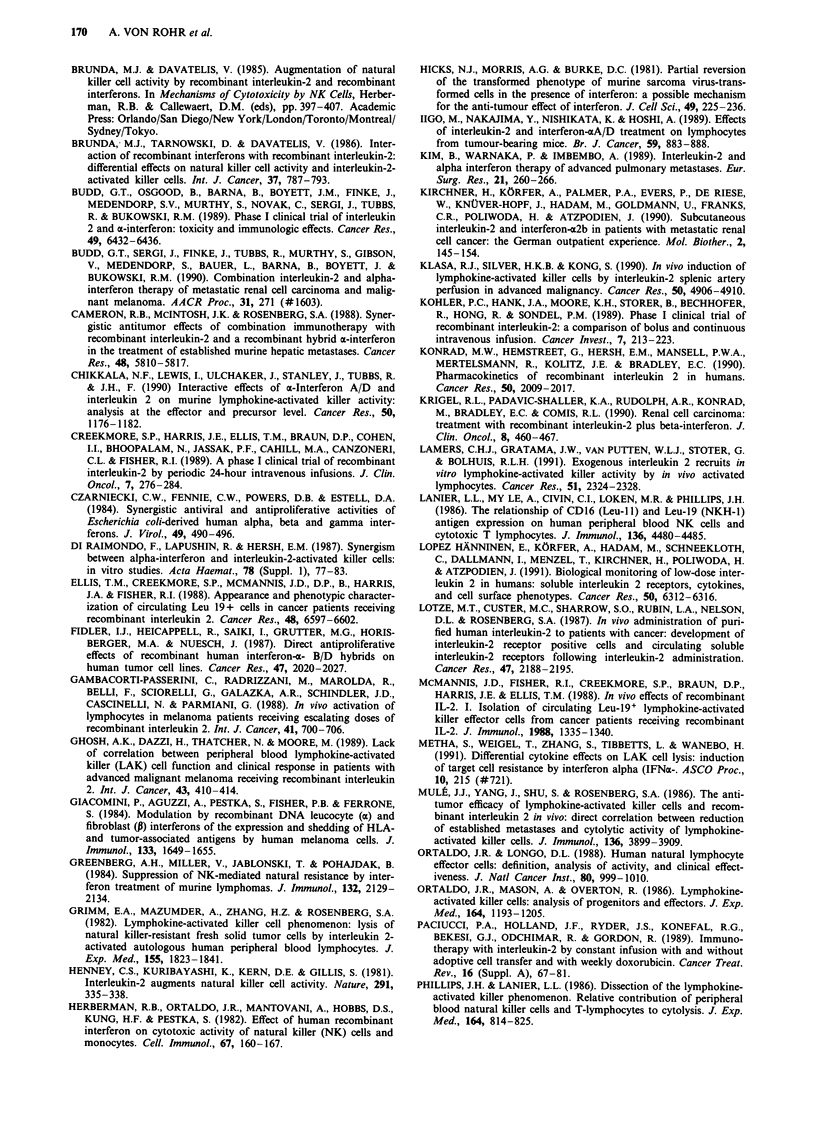

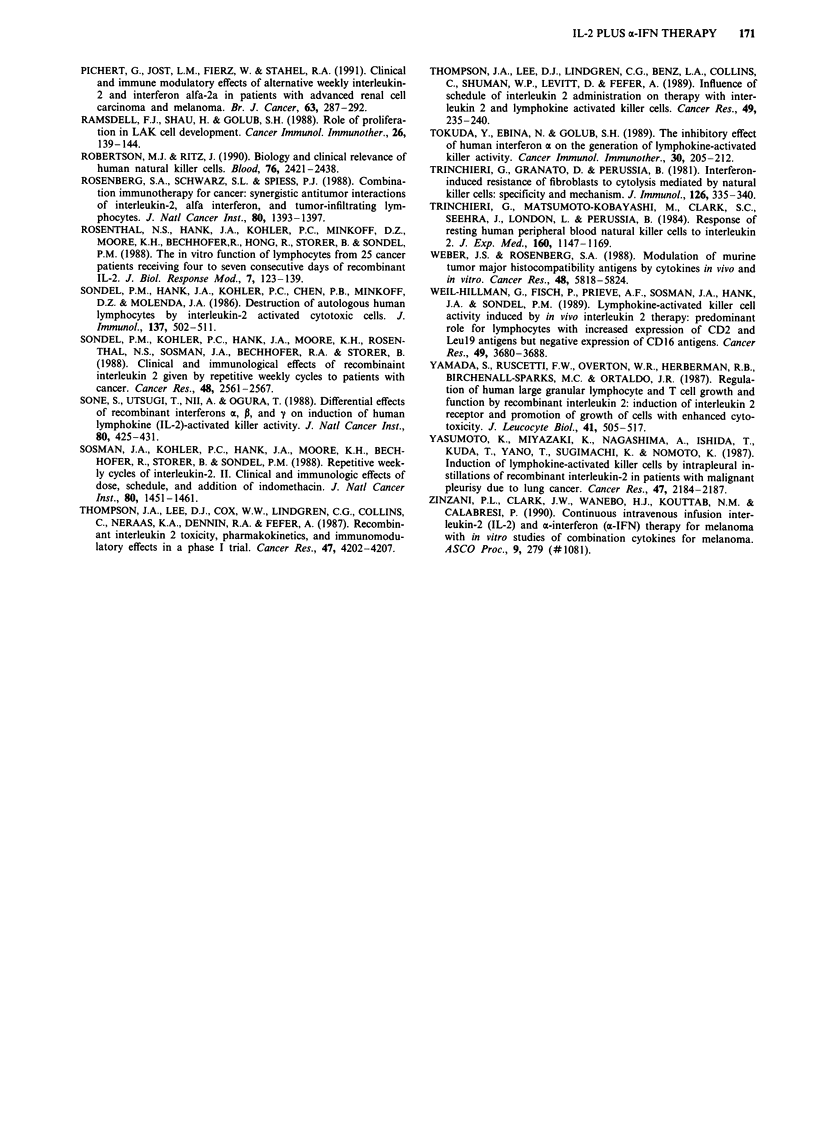


## References

[OCR_01104] Atkins M. B., Gould J. A., Allegretta M., Li J. J., Dempsey R. A., Rudders R. A., Parkinson D. R., Reichlin S., Mier J. W. (1986). Phase I evaluation of recombinant interleukin-2 in patients with advanced malignant disease.. J Clin Oncol.

[OCR_01111] Bich-Thuy L. T., Lane H. C., Fauci A. S. (1986). Recombinant interleukin-2-induced polyclonal proliferation of in vitro unstimulated human peripheral blood lymphocytes.. Cell Immunol.

[OCR_01117] Blay J. Y., Bertoglio J., Fradelizi D., Chouaib S. (1989). Functional interactions of IL2 and TNF in the differentiation of LGL into LAK effectors.. Int J Cancer.

[OCR_01122] Brunda M. J., Bellantoni D., Sulich V. (1987). In vivo anti-tumor activity of combinations of interferon alpha and interleukin-2 in a murine model. Correlation of efficacy with the induction of cytotoxic cells resembling natural killer cells.. Int J Cancer.

[OCR_01139] Brunda M. J., Tarnowski D., Davatelis V. (1986). Interaction of recombinant interferons with recombinant interleukin-2: differential effects on natural killer cell activity and interleukin-2-activated killer cells.. Int J Cancer.

[OCR_01145] Budd G. T., Osgood B., Barna B., Boyett J. M., Finke J., Medendorp S. V., Murthy S., Novak C., Sergi J., Tubbs R. (1989). Phase I clinical trial of interleukin 2 and alpha-interferon: toxicity and immunologic effects.. Cancer Res.

[OCR_01159] Cameron R. B., McIntosh J. K., Rosenberg S. A. (1988). Synergistic antitumor effects of combination immunotherapy with recombinant interleukin-2 and a recombinant hybrid alpha-interferon in the treatment of established murine hepatic metastases.. Cancer Res.

[OCR_01166] Chikkala N. F., Lewis I., Ulchaker J., Stanley J., Tubbs R., Finke J. H. (1990). Interactive effects of alpha-interferon A/D and interleukin 2 on murine lymphokine-activated killer activity: analysis at the effector and precursor level.. Cancer Res.

[OCR_01173] Creekmore S. P., Harris J. E., Ellis T. M., Braun D. P., Cohen I. I., Bhoopalam N., Jassak P. F., Cahill M. A., Canzoneri C. L., Fisher R. I. (1989). A phase I clinical trial of recombinant interleukin-2 by periodic 24-hour intravenous infusions.. J Clin Oncol.

[OCR_01180] Czarniecki C. W., Fennie C. W., Powers D. B., Estell D. A. (1984). Synergistic antiviral and antiproliferative activities of Escherichia coli-derived human alpha, beta, and gamma interferons.. J Virol.

[OCR_01186] Di Raimondo F., LaPushin R., Hersh E. M. (1987). Synergism between alpha-interferon and interleukin-2-activated killer cells: in vitro studies.. Acta Haematol.

[OCR_01191] Ellis T. M., Creekmore S. P., McMannis J. D., Braun D. P., Harris J. A., Fisher R. I. (1988). Appearance and phenotypic characterization of circulating Leu 19+ cells in cancer patients receiving recombinant interleukin 2.. Cancer Res.

[OCR_01199] Fidler I. J., Heicappell R., Saiki I., Grutter M. G., Horisberger M. A., Nuesch J. (1987). Direct antiproliferative effects of recombinant human interferon-alpha B/D hybrids on human tumor cell lines.. Cancer Res.

[OCR_01203] Gambacorti-Passerini C., Radrizzani M., Marolda R., Belli F., Sciorelli G., Galazka A. R., Schindler J. D., Cascinelli N., Parmiani G. (1988). In vivo activation of lymphocytes in melanoma patients receiving escalating doses of recombinant interleukin 2.. Int J Cancer.

[OCR_01210] Ghosh A. K., Dazzi H., Thatcher N., Moore M. (1989). Lack of correlation between peripheral blood lymphokine-activated killer (LAK) cell function and clinical response in patients with advanced malignant melanoma receiving recombinant interleukin 2.. Int J Cancer.

[OCR_01217] Giacomini P., Aguzzi A., Pestka S., Fisher P. B., Ferrone S. (1984). Modulation by recombinant DNA leukocyte (alpha) and fibroblast (beta) interferons of the expression and shedding of HLA- and tumor-associated antigens by human melanoma cells.. J Immunol.

[OCR_01224] Greenberg A. H., Miller V., Jablonski T., Pohajdak B. (1984). Suppression of NK-mediated natural resistance by interferon treatment of murine lymphomas.. J Immunol.

[OCR_01230] Grimm E. A., Mazumder A., Zhang H. Z., Rosenberg S. A. (1982). Lymphokine-activated killer cell phenomenon. Lysis of natural killer-resistant fresh solid tumor cells by interleukin 2-activated autologous human peripheral blood lymphocytes.. J Exp Med.

[OCR_01237] Henney C. S., Kuribayashi K., Kern D. E., Gillis S. (1981). Interleukin-2 augments natural killer cell activity.. Nature.

[OCR_01242] Herberman R. B., Ortaldo J. R., Mantovani A., Hobbs D. S., Kung H. F., Pestka S. (1982). Effect of human recombinant interferon on cytotoxic activity of natural killer (NK) cells and monocytes.. Cell Immunol.

[OCR_01248] Hicks N. J., Morris A. G., Burke D. C. (1981). Partial reversion of the transformed phenotype of murine sarcoma virus-transformed cells in the presence of interferon: a possible mechanism for the anti-tumour effect of interferon.. J Cell Sci.

[OCR_01305] Hänninen E. L., Körfer A., Hadam M., Schneekloth C., Dallmann I., Menzel T., Kirchner H., Poliwoda H., Atzpodien J. (1991). Biological monitoring of low-dose interleukin 2 in humans: soluble interleukin 2 receptors, cytokines, and cell surface phenotypes.. Cancer Res.

[OCR_01258] Kim B., Warnaka P., Imbembo A. (1989). Interleukin-2 and alpha interferon therapy of advanced pulmonary metastases.. Eur Surg Res.

[OCR_01263] Kirchner H., Körfer A., Palmer P. A., Evers P., De Riese W., Knüver-Hopf J., Hadam M., Goldman U., Franks C. R., Poliwoda H. (1990). Subcutaneous interleukin-2 and interferon-alpha 2b in patients with metastatic renal cell cancer: the German outpatient experience.. Mol Biother.

[OCR_01271] Klasa R. J., Silver H. K., Kong S. (1990). In vivo induction of lymphokine-activated killer cells by interleukin-2 splenic artery perfusion in advanced malignancy.. Cancer Res.

[OCR_01275] Kohler P. C., Hank J. A., Moore K. H., Storer B., Bechhofer R., Hong R., Sondel P. M. (1989). Phase 1 clinical trial of recombinant interleukin-2: a comparison of bolus and continuous intravenous infusion.. Cancer Invest.

[OCR_01281] Konrad M. W., Hemstreet G., Hersh E. M., Mansell P. W., Mertelsmann R., Kolitz J. E., Bradley E. C. (1990). Pharmacokinetics of recombinant interleukin 2 in humans.. Cancer Res.

[OCR_01287] Krigel R. L., Padavic-Shaller K. A., Rudolph A. R., Konrad M., Bradley E. C., Comis R. L. (1990). Renal cell carcinoma: treatment with recombinant interleukin-2 plus beta-interferon.. J Clin Oncol.

[OCR_01293] Lamers H. J., Gratama J. W., van Putten W. L., Stoter G., Bolhuis R. L. (1991). Exogenous interleukin 2 recruits in vitro lymphokine-activated killer activity by in vivo activated lymphocytes.. Cancer Res.

[OCR_01299] Lanier L. L., Le A. M., Civin C. I., Loken M. R., Phillips J. H. (1986). The relationship of CD16 (Leu-11) and Leu-19 (NKH-1) antigen expression on human peripheral blood NK cells and cytotoxic T lymphocytes.. J Immunol.

[OCR_01253] Ligo M., Nakajima Y., Nishikata K., Hoshi A. (1989). Effects of interleukin-2 and interferon-alpha A/D treatment on lymphocytes from tumour-bearing mice.. Br J Cancer.

[OCR_01312] Lotze M. T., Custer M. C., Sharrow S. O., Rubin L. A., Nelson D. L., Rosenberg S. A. (1987). In vivo administration of purified human interleukin-2 to patients with cancer: development of interleukin-2 receptor positive cells and circulating soluble interleukin-2 receptors following interleukin-2 administration.. Cancer Res.

[OCR_01320] McMannis J. D., Fisher R. I., Creekmore S. P., Braun D. P., Harris J. E., Ellis T. M. (1988). In vivo effects of recombinant IL-2. I. Isolation of circulating Leu-19+ lymphokine-activated killer effector cells from cancer patients receiving recombinant IL-2.. J Immunol.

[OCR_01333] Mulé J. J., Yang J., Shu S., Rosenberg S. A. (1986). The anti-tumor efficacy of lymphokine-activated killer cells and recombinant interleukin 2 in vivo: direct correlation between reduction of established metastases and cytolytic activity of lymphokine-activated killer cells.. J Immunol.

[OCR_01340] Ortaldo J. R., Longo D. L. (1988). Human natural lymphocyte effector cells: definition, analysis of activity, and clinical effectiveness.. J Natl Cancer Inst.

[OCR_01345] Ortaldo J. R., Mason A., Overton R. (1986). Lymphokine-activated killer cells. Analysis of progenitors and effectors.. J Exp Med.

[OCR_01350] Paciucci P. A., Holland J. F., Ryder J. S., Konefal R. G., Bekesi G. J., Odchimar R., Gordon R. (1989). Immunotherapy with interleukin-2 by constant infusion with and without adoptive cell transfer and with weekly doxorubicin.. Cancer Treat Rev.

[OCR_01357] Phillips J. H., Lanier L. L. (1986). Dissection of the lymphokine-activated killer phenomenon. Relative contribution of peripheral blood natural killer cells and T lymphocytes to cytolysis.. J Exp Med.

[OCR_01365] Pichert G., Jost L. M., Fierz W., Stahel R. A. (1991). Clinical and immune modulatory effects of alternative weekly interleukin-2 and interferon alfa-2a in patients with advanced renal cell carcinoma and melanoma.. Br J Cancer.

[OCR_01371] Ramsdell F. J., Shau H., Golub S. H. (1988). Role of proliferation in LAK cell development.. Cancer Immunol Immunother.

[OCR_01376] Robertson M. J., Ritz J. (1990). Biology and clinical relevance of human natural killer cells.. Blood.

[OCR_01380] Rosenberg S. A., Schwarz S. L., Spiess P. J. (1988). Combination immunotherapy for cancer: synergistic antitumor interactions of interleukin-2, alfa interferon, and tumor-infiltrating lymphocytes.. J Natl Cancer Inst.

[OCR_01386] Rosenthal N. S., Hank J. A., Kohler P. C., Minkoff D. Z., Moore K. H., Bechhofer R., Hong R., Storer B., Sondel P. M. (1988). The in vitro function of lymphocytes from 25 cancer patients receiving four to seven consecutive days of recombinant IL-2.. J Biol Response Mod.

[OCR_01393] Sondel P. M., Hank J. A., Kohler P. C., Chen B. P., Minkoff D. Z., Molenda J. A. (1986). Destruction of autologous human lymphocytes by interleukin 2-activated cytotoxic cells.. J Immunol.

[OCR_01401] Sondel P. M., Kohler P. C., Hank J. A., Moore K. H., Rosenthal N. S., Sosman J. A., Bechhofer R., Storer B. (1988). Clinical and immunological effects of recombinant interleukin 2 given by repetitive weekly cycles to patients with cancer.. Cancer Res.

[OCR_01406] Sone S., Utsugi T., Nii A., Ogura T. (1988). Differential effects of recombinant interferons alpha, beta, and gamma on induction of human lymphokine (IL-2)-activated killer activity.. J Natl Cancer Inst.

[OCR_01414] Sosman J. A., Kohler P. C., Hank J. A., Moore K. H., Bechhofer R., Storer B., Sondel P. M. (1988). Repetitive weekly cycles of interleukin-2. II. Clinical and immunologic effects of dose, schedule, and addition of indomethacin.. J Natl Cancer Inst.

[OCR_01419] Thompson J. A., Lee D. J., Cox W. W., Lindgren C. G., Collins C., Neraas K. A., Dennin R. A., Fefer A. (1987). Recombinant interleukin 2 toxicity, pharmacokinetics, and immunomodulatory effects in a phase I trial.. Cancer Res.

[OCR_01425] Thompson J. A., Lee D. J., Lindgren C. G., Benz L. A., Collins C., Shuman W. P., Levitt D., Fefer A. (1989). Influence of schedule of interleukin 2 administration on therapy with interleukin 2 and lymphokine activated killer cells.. Cancer Res.

[OCR_01432] Tokuda Y., Ebina N., Golub S. H. (1989). The inhibitory effect of human interferon alpha on the generation of lymphokine-activated killer activity.. Cancer Immunol Immunother.

[OCR_01437] Trinchieri G., Granato D., Perussia B. (1981). Interferon-induced resistance of fibroblasts to cytolysis mediated by natural killer cells: specificity and mechanism.. J Immunol.

[OCR_01441] Trinchieri G., Matsumoto-Kobayashi M., Clark S. C., Seehra J., London L., Perussia B. (1984). Response of resting human peripheral blood natural killer cells to interleukin 2.. J Exp Med.

[OCR_01447] Weber J. S., Rosenberg S. A. (1988). Modulation of murine tumor major histocompatibility antigens by cytokines in vivo and in vitro.. Cancer Res.

[OCR_01452] Weil-Hillman G., Fisch P., Prieve A. F., Sosman J. A., Hank J. A., Sondel P. M. (1989). Lymphokine-activated killer activity induced by in vivo interleukin 2 therapy: predominant role for lymphocytes with increased expression of CD2 and leu19 antigens but negative expression of CD16 antigens.. Cancer Res.

[OCR_01460] Yamada S., Ruscetti F. W., Overton W. R., Herberman R. B., Birchenall-Sparks M. C., Ortaldo J. R. (1987). Regulation of human large granular lymphocyte and T cell growth and function by recombinant interleukin 2: induction of interleukin 2 receptor and promotion of growth of cells with enhanced cytotoxicity.. J Leukoc Biol.

[OCR_01468] Yasumoto K., Mivazaki K., Nagashima A., Ishida T., Kuda T., Yano T., Sugimachi K., Nomoto K. (1987). Induction of lymphokine-activated killer cells by intrapleural instillations of recombinant interleukin-2 in patients with malignant pleurisy due to lung cancer.. Cancer Res.

